# Path Loss Prediction Model of 5G Signal Based on Fusing Data and XGBoost—SHAP Method

**DOI:** 10.3390/s25175440

**Published:** 2025-09-02

**Authors:** Tingting Xu, Nuo Xu, Jay Gao, Yadong Zhou, Haoran Ma

**Affiliations:** 1 School of Computer Science and Technology, Chongqing University of Posts and Telecommunications, Chongqing 400065, China; s241201040@stu.cqupt.edu.cn (N.X.); 2022214168@stu.cqupt.edu.cn (H.M.); 2 Key Laboratory of Big Data Intelligent Computing, Chongqing University of Posts and Telecommunications, Chongqing 400065, China; 3 School of Science, the University of Auckland, Auckland 1010, New Zealand; jg.gao@auckland.ac.nz; 4 China Unicom, Chongqing 400000, China; zhouyd@chinaunicom.cn

**Keywords:** 5G signal path loss, multimodal data feature fusion, XGBoost, machine learning

## Abstract

The accurate prediction of path loss is essential for planning and optimizing communication networks, as it directly impacts the user experience. In 5G signal propagation, the mix of varied terrain and dense high-rise buildings poses significant challenges. For example, signals are more prone to multipath effects and occlusion and shadowing occur often, leading to high nonlinearities and uncertainties in the signal path. Traditional and shallow models often fail to accurately depict 5G signal characteristics in complex terrains, limiting the accuracy of path loss modeling. To address this issue, our research introduces innovative feature engineering and prediction models for 5G signals. By utilizing smartphones as signal receivers and creating a multimodal system that captures 3D structures and obstructions in the N1 and N78 bands in China, the study aimed to overcome the shortcomings of traditional linear models, especially in mountainous areas. It employed the XGBoost algorithm with Optuna for hyperparameter tuning, improving model performance. After training on real 5G data, the model achieved a breakthrough in 5G signal path loss prediction, with an R^2^ of 0.76 and an RMSE of 3.81 dBm. Additionally, SHAP values were employed to interpret the results, revealing the relative impact of various environmental features on 5G signal path loss. This research enhances the accuracy and stability of predictions and offers a technical framework and theoretical foundation for planning and optimizing wireless communication networks in complex environments and terrains.

## 1. Introduction

Fifth-generation mobile communication technology (5G) is transforming wireless networks. It provides high bandwidth, low latency, and more device connections [1,2]. ITU-R M.2083 [3] states that “5G systems will give users seamless connectivity. They will also meet the needs of enhanced mobile broadband (eMBB), ultra-reliable low-latency communication (URLLC), and massive machine-type communication (mMTC). This shift is accelerating the deployment of 5G networks. Better accuracy in wireless propagation modeling is crucial for this development.” 5G has evolved into a widely adopted and relatively mature communication technology, particularly in the sub-6 GHz frequency bands, which support the majority of current commercial deployments and everyday mobile applications. Although millimeter-wave (mmWave) [4] bands are gradually being commercialized to unlock higher data rates and capacity, sub-6 GHz remains the backbone of current 5G networks due to its broader coverage and stability [5]. Due to the complex terrain and topographic variability in mountainous regions, even 4G networks have not yet achieved full coverage in many such areas. This underscores the necessity for more efficient and accessible methods to predict signal attenuation, enabling faster and more accurate deployment of 5G infrastructure. This research provides valuable insights for understanding signal propagation characteristics not only in sub-6 GHz bands but also in higher-frequency regimes such as millimeter waves, thereby offering exploratory value for the development of future 6G networks.

During network planning, path loss models estimate the attenuation of wireless signals in various environments. This estimation affects where base stations are placed, how spectra is used, and how coverage is optimized. The path loss prediction of 5G traditionally includes two types of models: deterministic models [6] and empirical models [7]. Deterministic and empirical models each have their strengths and limitations: the former excels in accuracy, but incurs high computational costs, while the latter offers computational efficiency at the expense of reduced adaptability in complex environments [8]. Due to constraints in computational resources and deployment costs, empirical models are more commonly adopted in practical 5G network deployments [9]. The 3GPP TR 38.901 path loss model as an empirical model is popular. It covers many scenarios, including urban macrocells, urban microcells, indoor offices, rural macrocells, and indoor factories. It works for both line-of-sight and non-line-of-sight conditions [10]. Shabbir et al. tested how well these models work in standard condition [11]. However, as 5G expands into urban areas and complex terrains, traditional models often struggle to provide the flexibility and accuracy needed, making it challenging to precisely model signal propagation.

Signal interference plays a significant role in influencing the accuracy and reliability of 5G signal path loss models [12]. Unlike traditional wireless systems, 5G networks operate in increasingly congested spectral environments, where co-channel interference, adjacent channel interference, and inter-symbol interference pose substantial challenges [13,14]. These interferences introduce fluctuations and distortions in the received signal strength, complicating the task of accurate path loss prediction [15]. In particular, the adoption of dense small-cell deployments and heterogeneous network architectures exacerbates interference effects, leading to highly variable propagation conditions [16]. Several studies have highlighted that interference is inherently stochastic and environment-dependent, making it difficult to model deterministically or to eliminate through conventional mitigation techniques [17,18]. Advanced signal processing and machine learning approaches have been proposed to address interference effects, yet these methods often require extensive training data and are limited by generalizability across different deployment scenarios [19,20].

To enhance empirical modeling, numerous researchers have explored the application of simulations and deep learning techniques. These methods help model signal patterns and identify key geometric features. Simulations show improvements of 30% to 43% over traditional methods [21]. However, they still struggle with real-world accuracy. Decision tree algorithms, such as XGBoost (extreme gradient boosting) and LightGBM (light gradient boosting machine), are more effective for path loss prediction [22]. They handle nonlinear data effectively. Some studies suggest power optimization strategies with dynamic coordination among power control agents. This approach aims to ensure coverage and capacity while reducing interference [23]. Yazici et al. compared various machine learning methods, highlighting the challenges faced by traditional models [24]. Traditional formulas have difficulty at high altitudes, around obstructions, and in irregular urban layouts. They also overlook key factors such as multipath effects and terrain reflection [25]. To balance the simplicity and low computational cost of empirical models with the high accuracy of deterministic approaches, a selection of easily accessible physical and geometric features from the real environment can be incorporated to enhance empirical modeling with minimal data requirements. In this context, machine learning (ML) models emerge as a powerful alternative, capable of capturing complex nonlinear relationships between these input features and propagation characteristics, thereby significantly improving prediction performance without the need for exhaustive environmental modeling. Researchers are now working to combine high-resolution building data with field measurements of signal strength [26,27]. For instance, Ethier et al. found that features such as frequency and link distance are crucial for understanding signal loss based on data from the UK and Canada [28]. This processing improves the model’s ability to represent geographical and radio frequency characteristics, providing robust data for XGBoost modeling. However, correlated features can limit the model’s performance. When adding new features, improvements are often marginal due to redundancy and data sparsity. To address this, we incorporate geographical and environmental factors, such as terrain elevation and vegetation index, to improve the feature set.

Unlike many existing studies that rely on idealized [28] or small-scale [25] datasets, our work addresses the lack of large, diverse datasets collected under complex mountainous terrain conditions. To overcome this limitation, we conducted extensive field measurements to obtain a sufficient quantity and variety of features relevant to real-world deployment scenarios. The challenges lie not only in data collection but also in the subsequent data processing and feature engineering, where identifying the key environmental and network factors affecting signal propagation in such irregular terrains remains a significant technical barrier. This contributes to the novelty of our approach and highlights the practical complexity addressed in this study.

Our study offers new insights by focusing on 5G signal path loss prediction in complex mountainous environments, which are often overlooked in existing literature dominated by urban or flat-terrain scenarios. Unlike prior studies that relied on limited or simulated datasets, we constructed a real-world dataset collected via UAV-assisted measurements in diverse terrain conditions. By incorporating a rich set of environmental and deployment features—such as altitude, vegetation index, building density, and base station parameters—we conducted a comprehensive feature analysis to identify key factors influencing signal attenuation. The proposed model demonstrates improved predictive performance and provides practical guidance for 5G network deployment in non-urban regions.

In addition, by employing a multimodal feature approach to gain a deeper understanding of what affects 5G signal loss in a complex environment, we combine SHAP (Shapley additive explanations) to interpret the model results. SHAP is a method used to explain the prediction results of machine learning models. This method has been widely applied in various fields, such as coronary heart disease prediction and credit default modeling.

Building on the above context, we propose a path loss prediction framework that integrates XGBoost for regression modeling and SHAP for model interpretability. To enhance model performance, we conducted hyperparameter tuning for the XGBoost algorithm using Optuna 4.4.0, a Bayesian optimization framework. The tuning process included key parameters such as learning rate, maximum tree depth, and the number of estimators. We used mobile phones as terminals to collect data [29]. To enhance accuracy and generalization, we employed a structured feature engineering approach before training. Given the periodic nature of directional data in wireless communication, we applied sine and cosine transformations to features such as base station azimuth and user angles, which reduces bias due to angular discontinuities. We also used one-hot encoding for categorical variables such as NR (NewRadio)_BAND and Base_Bandwidth to prevent information loss. We also applied Z-score normalization [30] to distance and send power features to ensure stable training. To capture the nonlinear aspects of signal propagation, we created high-order variables, including squared and reciprocal terms. This model is tailored for urban settings with complex terrain, taking into account factors such as topography, obstructions, and multipath effects. It enhances predictions of wireless signal characteristics, facilitating more accurate base station placement and improved network coverage in challenging areas. The findings of this research offer practical value for deploying 5G networks in mountainous cities, thereby enhancing user experiences and reducing planning costs.

This paper proposes a path loss prediction framework that integrates XGBoost for regression and SHAP for interpretability, effectively capturing the nonlinear propagation characteristics of 5G signals in urban environments. A structured feature set was constructed by incorporating physical, environmental, and network parameters through directional transformations, high-order terms, and categorical encodings. Hyperparameter tuning was performed via Bayesian optimization using Optuna to enhance model performance. The framework was validated using real-world measurements from mountainous urban areas, demonstrating its effectiveness in supporting 5G network planning.

## 2. Materials and Methods

In our measurement setup, the UAV maintained a stable horizontal flight posture throughout the data collection process, ensuring minimal variation in the antenna orientation of the mobile phone and thus reducing potential polarization mismatch effects. To further account for the influence of polarization mismatches and directional signal reception, the model incorporates features such as the distance to the base station, the mechanical downtilt, and the electrical downtilt of the base station antenna. These features enable the model to implicitly learn and compensate for polarization-related signal variations, as evidenced by the trends observed in the partial dependence plot (PDP), which confirm that such factors have a measurable impact on the received signal strength in our dataset.

**Figure 1:**
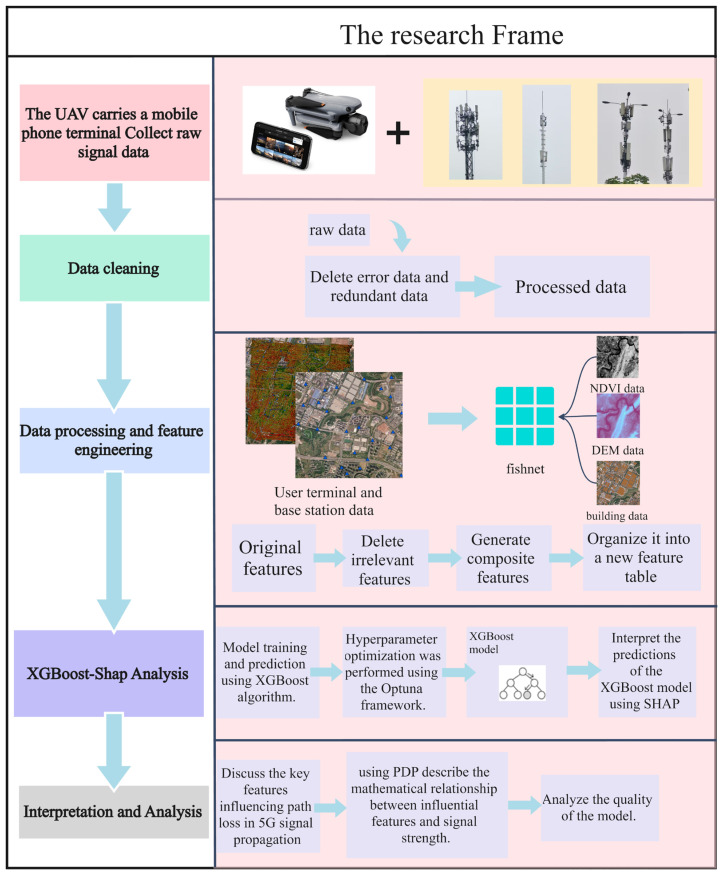
Workflow for constructing a multimodal 5G signal path loss prediction model.

In Figure 1, the whole workflow includes the collection of raw data by using the mobile phone carried by the UAV, and then the data cleaning of the raw data, including the deletion of erroneous data and redundant data. Next, data engineering is carried out on the data, and the processed user data and the base station data provided by the operator are combined with normalized difference vegetation index (NDVI) data, digital elevation model (DEM), data and building data for feature fusion and deletion of irrelevant features. Then, the existing features are generated for feature generation and all the processed features are integrated into fishnets. The data were imported into the XGBoost–SHAP model for analysis. XGBoost was used for model training, the Optuna framework was used for hyperparameter tuning, and then SHAP was used for interpretability analysis. Finally, the PDP was used to linearly analyze the key features that affect the model and then explain which main features affect the signal propagation.

The prediction task in this study was formulated by constructing an input feature set that integrates multiple categories of information. Base station parameters include, for example, antenna type, downtilt angle, and center frequency. User terminal parameters incorporate measurements such as download speed and measurement altitude. Environmental parameters consist of indicators such as the normalized difference vegetation index (NDVI) and digital elevation model (DEM) data. Composite and derived features are generated through systematic feature engineering, enabling the model to capture higher-order relationships and complex interactions among the original variables. The target variable for prediction is defined exclusively as the reference signal received power (RSRP) in a specific cell grid, thereby ensuring the integrity of the modeling process.

### 2.1. Data Collection and Feature Construction

**Figure 2:**
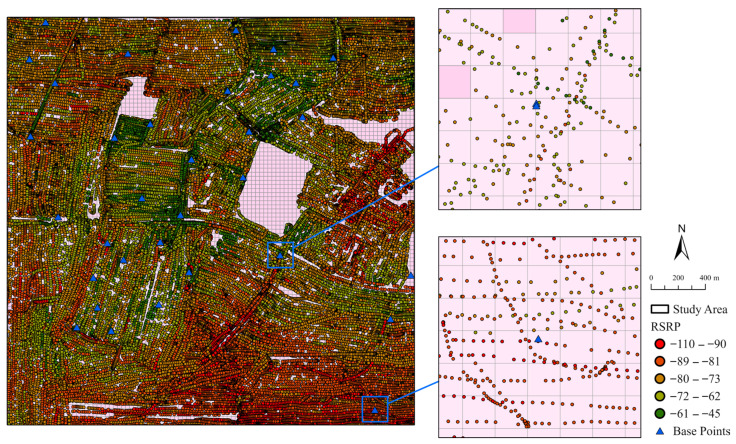
Research signal data (represented by green and red dots, where signal strength gradually increases from red to green) and base station data (represented by blue triangles) within a 3 km × 3 km area. These data points are mapped onto a vector grid with a resolution of 30 m as the computing unit.

The study area encompassed a square region measuring 3 km by 3 km surrounding the graduate dormitories of Chongqing University of Posts and Telecommunications, located in Yinglong Town, Nan’an District, Chongqing (Figure 2). Field measurements of 5G signals were collected, including reference signal received power (RSRP), reference signal received quality (RSRQ), signal-to-interference-plus-noise ratio (SINR), the elevation of measurement points, and corresponding base station cell global identity (CGI) data. Sampling was conducted using a smartphone equipped with the professional network testing tool Cellular Pro (developed by alibaba1126, Haidian District, Beijing, China), which was mounted on a drone. A total of 58,771 measurement points and 174 base station points were recorded. The smartphone utilized a Snapdragon X60 5G (developed by Qualcomm Incorporated, San Diego, CA, USA) modem as its baseband processor, and the data were integrated with macrocell data provided by China Unicom. Figure 2 illustrates the distribution of collected signal point data and computing unit cells.

Table 1 presents the features used in the data fusion. For example, NDVI_center represents NDVI data extracted at the center of each fishnet grid. Building_Coverage and Weighted_Height indicated building coverage information. SPEED_M_s_, SS_RSRP, and similar fields represent user terminal attributes, while Base_LONGITUDE, Base_Power, and related features characterize base station properties. Additionally, Match_Dist, Match_Angle, and other similar features describe the spatial relationships between user terminals and base stations.

Base station information: This includes the distance, height difference, azimuth, downtilt, and transmission power between the measurement point and the serving base station. Complete and accurate data were obtained through technical collaboration with the network operator.

Terrain features (DEM): The 30-m resolution digital elevation model data FathomDEM v1.0 for Eurasia and Africa [31] were obtained from the open data platform Zenodo.

Normalized difference vegetation index (NDVI): The NDVI data were calculated using Landsat images and the following formula:

(1) NDVI = (near-infrared − red)/(near-infrared + red)

Using the Google Earth Engine (GEE) cloud computing platform, all available Landsat 5/7/8/9 satellite images over the course of a year were processed by applying cloud and shadow removal techniques to obtain valid observations. The NDVI was calculated for each valid Landsat observation, and the annual maximum NDVI for each pixel was determined using linear interpolation and Savitzky–Golay (S–G) smoothing. The resulting dataset had a spatial resolution of 30 m and an annual temporal resolution [32].

Building information: The building data were also obtained from a global dataset hosted on the Zenodo platform, which includes both building footprints and height information. These data were generated using multisource remote sensing data and machine learning techniques. The dataset is provided in SHP format and utilizes the WGS84 coordinate system, offering global coverage, including China. The latest version (V4), released in 2025, provides improved accuracy and completeness compared to OpenStreetMap (OSM) data. Despite its high quality, some data gaps and distortions were identified; therefore, field surveys were conducted to verify and supplement the distorted building records [33].

The height of each measurement point above ground level was obtained by subtracting the corresponding ground elevation value from the digital elevation model (DEM) data from the altitude recorded by the mobile phone. This approach ensured a consistent and terrain-adjusted height reference for all measurements, particularly important in the complex topography of mountainous regions.

### 2.2. Feature Fusion and Engineering

**Figure 3:**
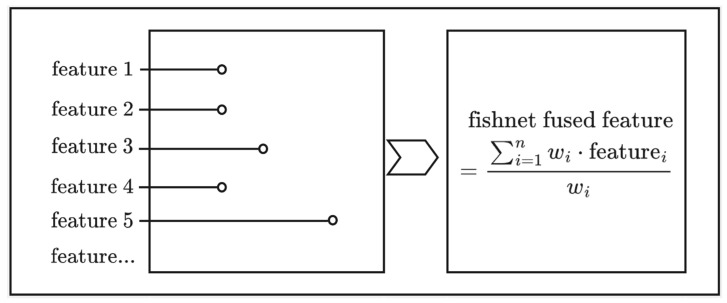
Discrete features are integrated into the fishnet features through weighted averaging, majority voting, or central value extraction methods.

As Figure 3 shows, most features were averaged using weights, while some features were determined by the mode or by taking the central point. All features were initially projected into a standard spatial reference system (WGS 1984/UTM Zone 48N). Within the 3 km × 3 km study area, we established a regular vector grid with a resolution of 30 m × 30 m, resulting in a total of 10,000 cells, to integrate and process terminal data, base station data, and other multimodal information, perform feature engineering, and assign all results as attributes of the grid, making it convenient to export them as input data for the model. In Figure 3, since the data points within each cell are not unique, we assigned the continuous variables of multiple points to the cells using a weighted average approach, while the categorical labels of these points were assigned based on the mode. The absence of data in certain cells is attributed to signal interference and specific areas (such as prisons) that restrict signal collection. To address this, we employed spatial interpolation combined with feature convolution generation to fill in the missing data. We then aggregated feature statistics at the grid level to create sample units for model training. To account for the periodic nature of directional features, we utilized dual-channel encoding with sine and cosine functions for angular variables. This Z-score normalization method effectively reduced discontinuities at boundary values, such as between 0° and 360°. For categorical variables, we implemented targeted encoding strategies to enhance their representation in the model. Continuous features related to distance and power were transformed using logarithmic functions and subsequently binned into intervals. This approach enabled us to capture nonlinear relationships and improve feature flexibility and model adaptability.

To address spatial incompleteness in the original data, we used spatial interpolation and propagation-based imputation techniques. We employed a step-by-step approach to fill in the missing spatial features. This involved multipass traversal and matrix-based diffusion. Additionally, we applied a 5 × 5 convolutional kernel to create weighted averages of building coverage within each grid cell. This ensured structural completeness and spatial consistency in the final training dataset.

Since raw features had inconsistent dimensionality, we standardized all input variables before modeling. To reduce the negative effects of redundancy and multicollinearity on model performance, we used SHAP for feature importance evaluation. Following the unified framework by Lundberg and Lee, SHAP quantifies the average marginal contribution of each feature across all combinations. This enables a consistent approach to model interpretability and feature selection. We kept only variables that significantly affected signal strength prediction for model construction [34].

### 2.3. Model Construction and Training

The XGBoost algorithm employed in this study is an ensemble boosting tree method capable of effectively modeling nonlinear relationships and interactions among features. It demonstrates strong generalization ability and has been widely applied in both regression and classification tasks [35].

Model representation:

(2) $${\hat{y}}_{i}=\sum_{k=1}^{K}f_{k}(x_{i}),f_{k}\in F$$

To efficiently search and optimize the hyperparameters of the XGBoost regression model, this study adopted the Optuna framework for automated Bayesian optimization. Optuna is based on the tree-structured Parzen estimator (TPE) sampling algorithm and offers advantages such as high efficiency, flexibility, and support for distributed optimization, making it well suited for improving model performance [36]. Specifically, we designed an objective function that defines a hyperparameter search space to be optimized. This space includes, but is not limited to, the following parameters: learning rate (learning_rate), maximum tree depth (max_depth), number of leaves (max_leaves), tree growth policy (grow_policy), column sampling rates (colsample_bytree, colsample_bylevel, colsample_bynode), subsample ratio (subsample), minimum child weight (min_child_weight), regularization parameters (reg_alph, reg_lambda), and the number of trees (n_estimators).

Objective function:

(3) $$\theta^{*}=arg\;\underset{\theta \in H}{max\;}R^{2}(\theta )$$

During the hyperparameter optimization process, in each iteration (trial), Optuna’s tree-structured Parzen estimator (TPE) sampler selects a set of hyperparameter values from the defined search space, under which an XGBoost model is trained. Subsequently, 5-fold cross-validation is conducted to evaluate the model’s performance, and the average coefficient of determination (R^2^) obtained from cross-validation is used as the metric to guide Optuna’s subsequent hyperparameter sampling and search.

Core idea of TPE (tree-structured Parzen estimator):

(4) $$\theta^{*}=arg\;\underset{\theta \in H}{max}\frac{l(\theta )}{g(\theta )}$$

To accelerate the training process and fully utilize hardware resources, all model training and evaluation in this experiment was conducted with GPU acceleration enabled. Ultimately, Optuna records and returns the hyperparameter combination that achieves the optimal cross-validation R^2^ score, based on which the final model is trained on the entire training dataset. This model is then serialized and saved to serve as the foundation for subsequent interpretability analyses, such as SHAP explanations.

This hyperparameter tuning process not only enables efficient exploration of the high-dimensional hyperparameter space but also ensures that the final model achieves good fit and generalization performance on the training set.

## 3. Results

This section presents the experimental results of 5G signal path loss modeling based on multimodal environmental features. The model’s performance is evaluated from multiple perspectives, including the coefficient of determination (R^2^), mean squared error (MSE), root mean squared error (RMSE), and mean absolute error (MAE).

### 3.1. Model Performance Metrics

**Figure 4:**
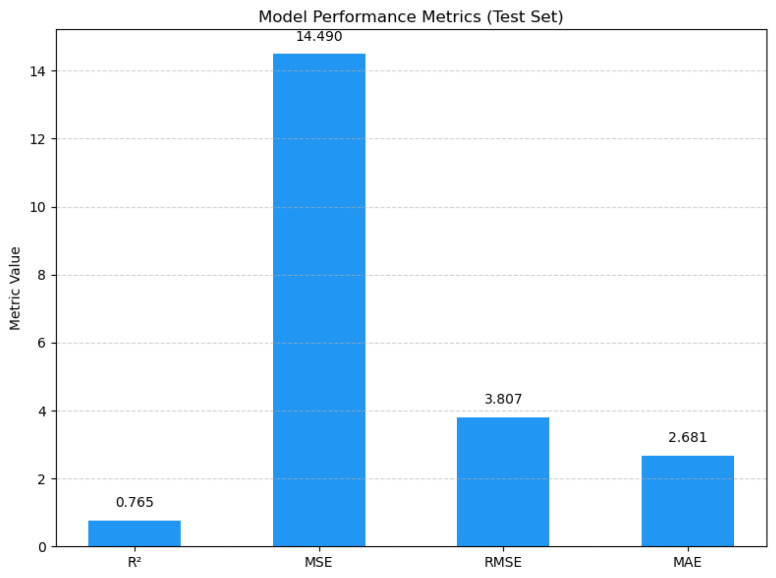
Model evaluation metrics on the test set.

This paper provides a comprehensive evaluation of the proposed path loss prediction model using various performance metrics. Figure 4, showing the model performance on the test set, is summarized as follows.

Coefficient of determination (R^2^): The model achieved an R^2^ value of 0.7647, indicating that approximately 76.47% of the variance in the target variable was explained. This demonstrates strong fitting capability and good predictive performance.

MSE and RMSE: The MSE was 14.4904 dBm, indicating a low average squared difference between predicted and actual values, which suggests minimal prediction errors. The RMSE was 3.8066 dBm, indicating that the absolute error in predicting signal strength was controlled within approximately 3.9 dBm. This value falls within the acceptable error range in practical communication engineering, which typically ranges from 3 to 5 dBm.

MAE: The MAE was 2.6813 dBm, which further confirmed the model’s stability and accuracy in its prediction.

These results demonstrate that the proposed model effectively captures the attenuation characteristics of wireless signals in complex mountainous urban environments, exhibiting high predictive accuracy and strong generalization capabilities.

After the model was tuned with Optuna, the R^2^ value increased from 0.65 to 0.7647. Table 2 shows the final XGBoost parameters after the tuning process.

### 3.2. Feature Importance Analysis

**Figure 5:**
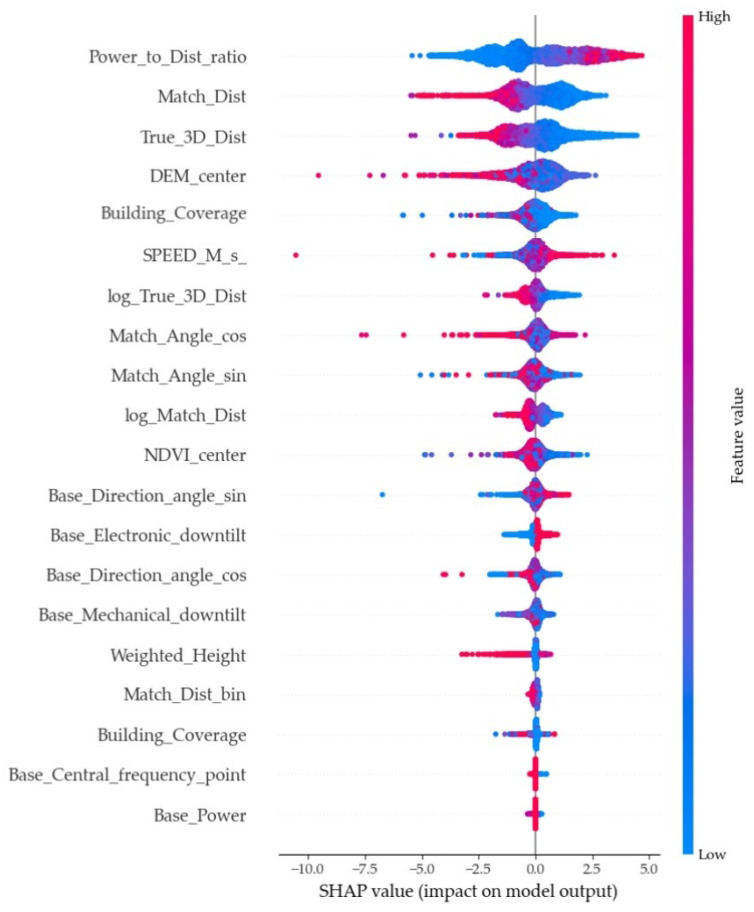
The features ranked from most to least significant in influencing the model.

SHAP is commonly used for evaluating feature importance. In this study, to further understand the model’s predictive capabilities, we employed the SHAP method to analyze feature importance and to identify which features primarily influenced signal strength. Figure 5 presents the distribution of SHAP values for the key features.

By calculating the average marginal contribution of each input feature to the model output (measured by the mean absolute SHAP value), the dependence of the model on different features was revealed (Figure 5). The analysis indicated that the model primarily relies on the following categories of features for path loss prediction.

First, propagation-related variables are central to modeling wireless signal behavior. For instance, features such as Power_to_Dist_ratio, Match_Dist, True_3D_Dist, log_Match_Dist, and log_True_3D_Dist are derived through multimodal data fusion and directly reflect the influence of signal propagation distance and transmission power on received signal strength. Among these, Power_to_Dist_ratio—an integrated variable combining both power and distance effects—emerged as the most influential contributor to the model’s performance.

Spatial terrain information also plays a critical role in path loss prediction. Features including ALT_M_ (elevation of measurement points), DEM_center (representing terrain elevation), and NDVI_center (vegetation coverage at the grid center) significantly impact signal attenuation. These variables highlight that signal propagation is not only a function of horizontal distance but is also shaped by terrain-induced obstruction and scattering effects. This confirms the model’s ability to effectively incorporate and leverage geospatial characteristics.

In addition, structural environmental features further enrich the model’s representational capacity. Building_Coverage, which ranked in the top five in the results, quantifies impact of the density of urban structures and serves as a proxy for assessing local blockage conditions. Due to the ranking priority of its SHAP value, it can be known that the signal attenuation and diffraction caused by the obstruction of the building affect the linear relationship of the model regarding distance and frequency. It captures the effects of both line-of-sight and non-line-of-sight propagation without requiring explicit LOS/NLOS labels.

Directional and mobility-related features enhance the model’s responsiveness to dynamic communication contexts. Match_Angle_cos describes the alignment between base station coverage direction and user location, while SPEED_M_s_ reflects the mobility state of the user equipment. The model’s strong reliance on these features suggests its capacity to learn beam directionality and the effect of movement-induced channel variability, particularly under complex propagation scenarios.

The SHAP analysis results demonstrate that the constructed model not only learns the traditional path loss dependencies on distance and power but also effectively integrates multidimensional information such as spatial terrain, environmental structure, and directionality. This enables the model to establish a more generalizable and practically meaningful representation of signal attenuation in the feature space. Such capability allows the model to more accurately adapt to 5G signal propagation behaviors in complex urban scenarios, exhibiting strong interpretability and physical consistency.

### 3.3. Model Interpretability

Through partial dependence plot (PDP) analysis, we further interpreted the model’s prediction results. The plots revealed the trends of influence of key features on path loss.

**Figure 6:**
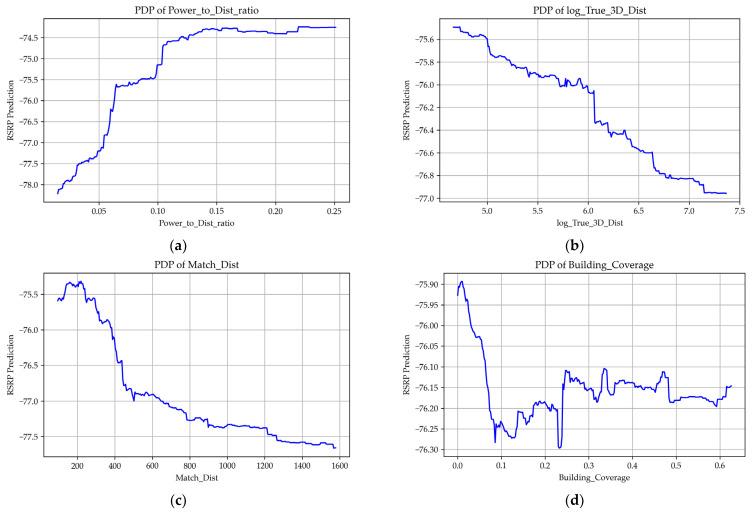

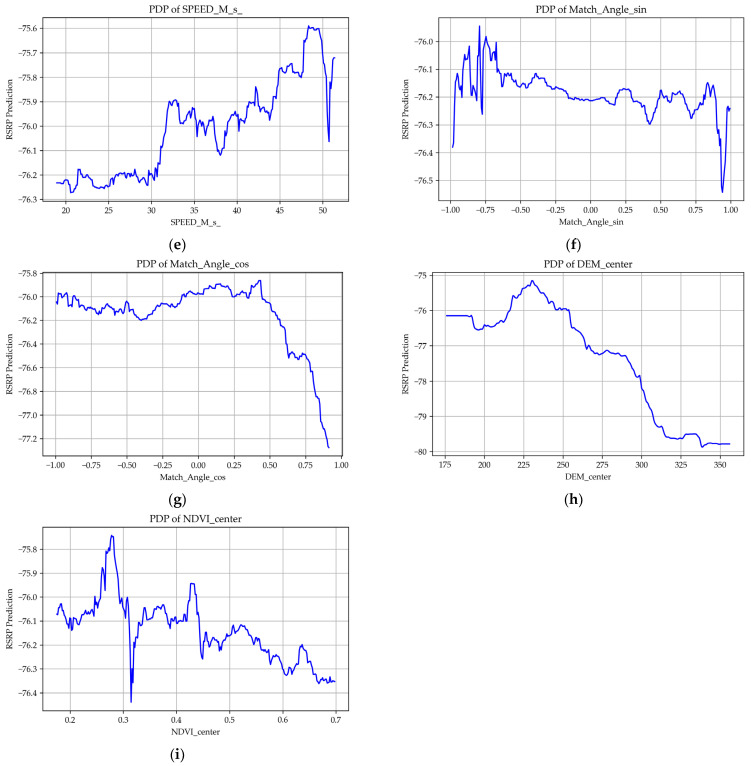
Influence factor graph. (**a**) Joint effect of transmission power and distance. (**b**) True distance between measurement point and base station calculated using Manhattan horizontal distance combined with vertical height difference. (**c**) Horizontal distance between measurement point and base station calculated by Manhattan distance. (**d**) Weighted building density around the measurement point. (**e**) Network speed at the measurement point. (**f**) Sine of the angle between base station and measurement point. (**g**) Cosine of the angle between base station and measurement point. (**h**) DEM value within a single grid cell. (**i**) Proportion of NDVI within a single grid cell.

As shown in Figure 6, signal strength exhibits a positive correlation with Power_to_instance_ratio and SPEED_M_s_, while it demonstrates an inverse relationship with True_3D_Dist, Match_Dist, Building_Coverage, Match_angle_cos, and DEM_center. Additionally, signal strength shows a fluctuating correlation with Match_angle_sin and NDVI_center. The Manhattan distance was used because the horizontal distance between some terminals and the base station reaches several kilometers, which leads to errors when using the Euclidean distance.

The Power_to_instance_ratio between the base station and the user terminal has a clearly positive effect on path loss. Specifically, signal strength increases significantly as the Power_to_instance_ratio rises from 0 to 0.1, and then gradually stabilizes in the range from 0.1 to 0.25.

True_3D_Dist exhibits a consistently strong inverse relationship with signal strength throughout its range, indicating that greater actual distance between the base station and user terminal leads to lower signal strength, which aligns well with physical expectations.

Building_Coverage also shows a notable inverse correlation with signal strength. A sharp decrease in signal strength occurs when the coverage increases from 0 to 0.05, suggesting that the presence or absence of building obstructions directly impacts signal quality. In denser built environments, signal attenuation becomes more pronounced due to obstruction effects between the measurement points and surrounding structures.

The sine of the angle between the base station and the measurement point (Match_angle_sin) displays a fluctuating correlation with signal strength: rising within the range of −1.0 to −0.75, and then gradually decreasing from −0.75 to 1.0. Conversely, Match_angle_cos remains relatively stable from −1.0 to 0.5, but demonstrates an inverse trend from 0.5 to 1.0.

Although DEM_center generally shows an inverse relationship with signal strength, a local positive correlation is observed within the elevation range of 175 to 225 m.

The NDVI_center feature exhibits a fluctuating inverse relationship with signal strength, with a particularly sharp drop observed around a value of 0.3. Although its linear correlation with signal strength is not prominent, vegetation density still exerts a measurable impact in densely vegetated areas.

## 4. Discussion

This research employed environmental characteristics and geographic data to develop an XGBoost regression model. The model was optimized through automated hyperparameter tuning facilitated by Optuna. By systematically refining the initial features, we observed a significant enhancement in the model’s predictive accuracy. To address periodic angular variables, sine and cosine transformations were utilized, thereby maintaining their cyclical properties and mitigating issues related to angular discontinuities. Categorical variables, such as frequency bands and base station bandwidths, are encoded to enable the model to effectively distinguish between different categories. Additionally, distance features are transformed logarithmically to correct for their skewed distributions. An interaction feature is introduced, which combines base station power and distance ratio, thereby enhancing the model’s physical relevance. The implementation of distance binning allows the model to capture nonlinear, piecewise patterns in signal attenuation. Collectively, these multidimensional feature transformations enrich the input data and integrate the physical principles of signal propagation, facilitating a more accurate representation of the nonlinear relationships between signal attenuation and the complexities of urban environments. This approach also improves both the stability and interpretability of predictions. The results corroborate previous research, indicating that gradient boosting trees are proficient in predicting wireless propagation. Notably, Shaibu et al. (2024) demonstrated the efficacy of XGBoost in path loss modeling, highlighting its ability to capture intricate, nonlinear relationships arising from building obstructions and multipath fading [37].

**Figure 7:**
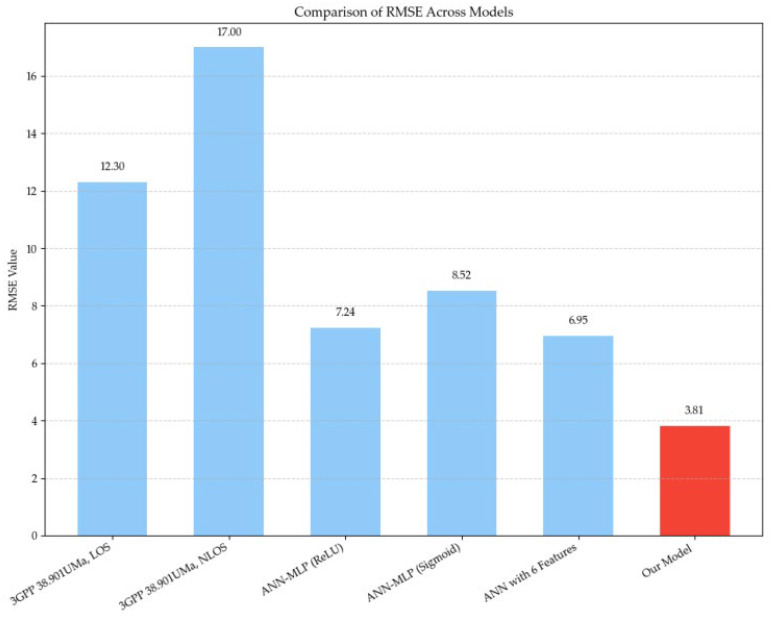
Comparison of RMSE across models [10,23,38].

In Table 3 and Figure 7, it can be observed that the proposed XGBoost-based prediction framework in this study achieves a substantially lower RMSE compared to other benchmark models, demonstrating its superior predictive capability. This performance gain can be attributed to the integration of multimodal features that comprehensively capture physical, environmental, and network-related factors influencing signal propagation. Furthermore, the adoption of systematic feature engineering—incorporating directional transformations, high-order interaction terms, and categorical encodings—enables the model to better represent the complex, nonlinear relationships inherent in 5G path loss. As a result, the proposed approach delivers more accurate and robust path loss predictions, offering tangible benefits for network planning and optimization, particularly in challenging urban and mountainous terrains.

Traditional model formula:

(5) $${PL}_{LOS}=28.0+22{log}_{10}(d_{3D})+20{log}_{10}(f_{c})$$

(6) $${PL}_{NLOS}=max({PL}_{LOS},28.0+13.54+39.08{log}_{10}(d_{3D})+20{log}_{10}(f_{c})-0.6(h_{UE}-1.5))$$

Moreover, the error level achieved in this study fells within the acceptable range for practical engineering applications. During the data collection process, various factors such as signal interference, environmental obstacles, and the type of equipment used can introduce uncertainty. According to existing research, the typical error range in signal strength prediction is approximately 3 to 5 dBm [39]. These inaccuracies underscore the need for robust models that can effectively generalize across varying spatial and environmental conditions. The results of this study align with this range, demonstrating the model’s practical utility.

The examination of SHAP and PDP data reveals a distinct linear correlation between signal loss and both the distance from the measurement point to the base station and the power levels involved. Additionally, significant associations with vegetation coverage and building density are observed. Although signal loss is not directly influenced by buildings within the current vector grid cell, it is affected by adjacent structures. Consequently, the feature labeled building_coverage exerts a more substantial influence on the model compared to the broader category of building_coverage. However, the impact of buildings on signal loss is less pronounced than anticipated, primarily due to the absence of explicit line of sight (LOS) and non-line of sight (NLOS) classifications. The data collection methodology employed drones, which precluded the verification of whether buildings obstructed the line of sight between measurement points and base stations. Given our focus on user-experienced signal loss in practical scenarios, it is impractical to assign ideal LOS/NLOS labels based on controlled experimental conditions.

The anomalous shape of the PDP curve, which indicates an initial increase in signal strength followed by a decrease with distance, contradicts the expected inverse relationship. This phenomenon can be attributed to signal blind zones located directly above and below the tower, which significantly influence the distribution of the curve and reflect the positioning of antennas on signal towers. Furthermore, the network speed curve displays oscillatory behavior rather than smooth trends, which we attribute to measurement delays inherent in the software utilized for capturing signal speed.

Despite these observations, the model’s R^2^ value has not surpassed 0.8, and there remains potential for improvement in the RMSE. This finding is consistent with prior research indicating that machine learning models often encounter challenges related to feature complexity and data quality. Kumar et al. (2020) emphasized that accurate predictions of wireless signal loss necessitate comprehensive feature representation [40], particularly in the absence of critical dynamic channel parameters such as temporal correlation and multipath delay spread. Additionally, noise and labeling inaccuracies within the measurement data constrain the model’s generalization capabilities, as noted by Zhao et al. (2019) [41].

To address these challenges, future research should enhance model performance by integrating significant features such as LOS/NLOS classification and path loss exponents. To improve model interpretability, methodologies like SHAP can clarify the impact of environmental factors on signal loss, thereby fostering a deeper understanding of wireless propagation and guiding more effective engineering decisions. Key considerations include building height, the distance between users and base stations, and the density and height of surrounding structures. These insights are essential for optimizing base station layouts and assessing signal coverage.

## 5. Conclusions

This paper proposes a 5G path loss model that integrates multimodal geographic environmental information with machine learning techniques. To address the common issue of insufficient feature dimensionality in existing research, heterogeneous features such as terrain elevation, vegetation index, and base station parameters are combined, and GIS tools are employed to achieve spatial visualization and a unified representation. This approach facilitates reliable data cleaning and feature fusion, significantly enhancing the expression and independence of feature dimensionality. During model development, a gradient boosting regression model based on XGBoost is introduced, complemented by SHAP for interpreting feature importance, thereby improving model explanation ability and decision transparency. The experimental results clearly demonstrate the superior performance of the proposed model. Our R^2^ achieved a value of 0.7647. RMSE was 3.8066 dBm. MAE was 2.6813, indicating a significant improvement in accurately in capturing real-world signal attenuation in complex urban environments. Such a substantial improvement not only validates the effectiveness of integrating environmental features and machine learning but also highlights the practical potential of this approach in guiding 5G network planning and optimization.

Experimental results demonstrate that the developed path loss model exhibits robust generalization capabilities and predictive accuracy in capturing the complex attenuation patterns of signal propagation. This approach offers theoretical support and technical guidance for base station site selection, network optimization, and resource allocation in mountainous urban areas, thereby holding substantial practical value. The XGBoost model effectively captures multidimensional nonlinear relationships in complex urban environments, demonstrating the potential of machine learning in wireless propagation while highlighting the current method limitations and identifying opportunities for future improvement. In terms of model interpretability, SHAP and similar techniques quantify the contributions of environmental variables to signal attenuation, thereby aiding in the understanding of wireless propagation mechanisms and supporting informed engineering decisions. Key factors such as building height, the distance between users and base stations, and the density and height distribution of buildings are identified as major influencers, providing direct guidance for optimizing base station layout and evaluating signal coverage. These outputs reveal that integrated heterogeneous multisource data, dynamic channel parameters, and more comprehensive feature sets, along with the fusion of physical propagation models and data-driven approaches, will improve the precise coverage and facilitate efficient deployment in intelligent communication networks.

Due to limitations in data collection via drones and mobile terminals, LOS and NLOS labels are difficult to obtain. Future research should optimize data acquisition methods and integrate real-time user trajectory data with dynamic propagation modeling to extend the model’s spatiotemporal adaptability and online optimization capabilities.

Although the use of a mobile phone as the measurement terminal may not achieve the same hardware-level accuracy as specialized channel sounding equipment, it reflects the performance and channel characteristics experienced by actual user equipment (UE) in operational networks. This choice enables the proposed model to capture real-world propagation effects, including antenna patterns, device hardware constraints, and environmental influences, particularly in complex mountainous terrain. The large-scale and diverse dataset collected ensures statistical robustness, making the results directly applicable to practical network planning and optimization tasks, while complementing rather than replacing high-precision laboratory measurements.

## Figures and Tables

**Table 1:** Engineered feature table.

Feature Type	Feature	Explanation
Environmental features	NDVI center	NDVI value at the geometric center of the grid cell, reflecting vegetation coverage.
	Building_Coverage	The proportion of the grid area covered by buildings, calculated from building footprints intersecting the grid.
	Weighted_Height	The average building height within the grid, weighted by the relative area of each building footprint.
	DEM center	The digital elevation model value at the center of the grid cell, representing local terrain elevation.
UE terminal features	SPEED_M_s_	Average downlink speed (in meters per second) received by user terminals within the grid.
	ALT_M_	Average altitude (in meters) of terminals located within the grid.
	NETWORK_TYPE	Predominant network configuration type (e.g., SA or NSA) observed among terminals in the grid.
	NR_TAC	Most frequent tracking area code (TAC) of base stations serving terminals in the grid.
	NR_BAND	Most commonly observed 5G NR frequency band (e.g., n71) among terminals in the grid.
	SS_RSRP	Average secondary synchronization reference signal received power measured by terminals in the grid.
	SS_RSRQ	Average reference signal received quality reported by terminals within the grid.
	SS_SINR	Average signal-to-interference-plus-noise ratio of terminals located in the grid area.
Base station features	Base_LONGITUDE	Longitude of base stations associated with terminals in the grid.
	Base_LATITUDE	Latitude of base stations associated with terminals in the grid.
	Base_Direction_angle	Azimuth angle (in degrees) of antennas of base stations serving terminals in the grid.
	Base_Central_frequency_point	Central frequency (in MHz) of carriers used by serving base stations.
	Base_Bandwidth	Bandwidth (in MHz) of base stations matched to terminals within the grid.
	Base_Electronic_downtilt	Electronic downtilt angle (in degrees) of antennas on serving base stations.
	Base_Mechanical_downtilt	Mechanical downtilt angle (in degrees) of base station antennas within the grid.
	Base_Power	Transmission power (in dBm) of base stations connected to terminals in the grid.
Integrated features	Match_Dist	Average Manhattan distance between terminals and their associated base stations.
	True_3D_Dist	3D Euclidean distance between terminals and base stations, calculated using horizontal distance and height difference.
	Match_Angle	Average horizontal angle between terminals and their serving base stations.

**Table 2:** Parameter values after optimization by Optuna.

Parameters	Value
n_estimators	1000
learning_rate	0.018085590088686893
max_depth	0
max_leaves	256
grow_policy	‘lossguide’
colsample_bylevel	1.0
colsample_bynode	0.75
colsample_bytree	0.75
min_child_weight	2
subsample	0.7
gamma	0.5827914682793354
reg_alpha	1.346006160434856
reg_lambda	5.654149254077154
objective	‘reg:squarederror’
verbosity	0
random_state	667

**Table 3:** Comparison of model performance metrics.

Models	RMSE	R^2^	MAE
3GPP 38.901UMa, LOS [10]	12.3		
3GPP 38.901UMa, NLOS [10]	17.0		
ANN-MLP (4-feature-based) [38]	8.40197	0.67463	6.47590
ANN-MLP (single-feature-based) [38]	8.61307	0.66113	6.58675
ANN model incorporating six composite features [28]	6.95		
Our Model	3.81	0.7647	2.6813

## Data Availability

The datasets generated and analyzed during the current study are available from the corresponding author upon reasonable request.
